# Grapevine *Rpv3*-, *Rpv10*- and *Rpv12*-mediated defense responses against *Plasmopara viticola* and the impact of their deployment on fungicide use in viticulture

**DOI:** 10.1186/s12870-021-03228-7

**Published:** 2021-10-14

**Authors:** Chantal Wingerter, Birgit Eisenmann, Patricia Weber, Ian Dry, Jochen Bogs

**Affiliations:** 1 State Education and Research Center of Viticulture, Horticulture and Rural Development, Neustadt/Weinstr, Germany; 2 Centre for Organismal Studies Heidelberg, University of Heidelberg, Heidelberg, Germany; 3 CSIRO Agriculture & Food, Urrbrae, SA 5064 Australia; 4 Technische Hochschule Bingen, 55411 Bingen am Rhein, Germany

**Keywords:** Disease resistance, Downy mildew, Grapevine, *Rpv12*, *Rpv10*, *Rpv3*, *Vitis vinifera*, Stilbenes, *Plasmopara viticola*, *avrRpv*

## Abstract

**Background:**

The high susceptibility of European grapevine cultivars (*Vitis vinifera*) to downy mildew (*Plasmopara viticola)* leads to the intensive use of fungicides in viticulture. To reduce this input, breeding programs have introgressed resistance loci from wild *Vitis* species into *V. vinifera*, resulting in new fungus-resistant grapevine cultivars (FRC). However, little is known about how these different resistance loci confer resistance and what the potential reduction in fungicide applications are likely to be if these FRCs are deployed. To ensure a durable and sustainable resistance management and breeding, detailed knowledge about the different defense mechanisms mediated by the respective *Rpv* (*Resistance to P. viticola*) resistance loci is essential.

**Results:**

A comparison of the resistance mechanisms mediated by the *Rpv3–1, Rpv10* and/or *Rpv12*-loci revealed an early onset of programmed cell death (PCD) at 8 hours post infection (hpi) in *Rpv12-*cultivars and 12 hpi in *Rpv10-*cultivars, whereas cell death was delayed in *Rpv3*-cultivars and was not observed until 28 hpi. These temporal differences correlated with an increase in the *trans*-resveratrol level and the formation of hydrogen peroxide shortly before onset of PCD. The differences in timing of onset of *Rpv*-loci specific defense reactions following downy mildew infection could be responsible for the observed differences in hyphal growth, sporulation and cultivar-specific susceptibility to this pathogen in the vineyard. Hereby, *Rpv3-* and *Rpv12/Rpv3-*cultivars showed a potential for a significant reduction of fungicide applications, depending on the annual *P. viticola* infection pressure and the *Rpv*-loci. Furthermore, we report on the discovery of a new *P. viticola* isolate that is able to overcome both *Rpv3-* and *Rpv12*-mediated resistance.

**Conclusion:**

This study reveals that differences in the timing of the defense reaction mediated by the *Rpv3-*, *Rpv10*- and *Rpv12*-loci, result in different degrees of natural resistance to downy mildew in field. Vineyard trials demonstrate that *Rpv12/Rpv3-* and *Rpv3*-cultivars are a powerful tool to reduce the dependence of grape production on fungicide applications. Furthermore, this study indicates the importance of sustainable breeding and plant protection strategies based on resistant grapevine cultivars to reduce the risk of new *P. viticola* isolates that are able to overcome the respective resistance mechanism*.*

**Supplementary Information:**

The online version contains supplementary material available at 10.1186/s12870-021-03228-7.

## Background

*Plasmopara viticola,* the causal agent of grapevine downy mildew, is one of the most economically important pathogens of grapevine. This host-specific oomycete pathogen is believed to have been introduced into Europe from North America in the late nineteenth century [1]. While North American and Asian *Vitis* species possess resistance loci against *P. viticola*, the cultivated European *Vitis vinifera* cultivars lack this genetic resistance, making them highly susceptible to this pathogen [1–3]. Consequently, viticulture is heavily dependent on the use of fungicides to prevention yield and quality losses, as demonstrated by the fact that more than 70% of the total quantity of fungicides used in Europe are applied in viticulture [4]. To reduce these ecological and economic burdens on wine production, several breeding programs have successfully introgressed resistance loci from wild North American and Asian *Vitis* species into *V. vinifera* resulting in new fungus-resistant grapevine cultivars (FRC) [5, 6]. While numerous FRCs are available for wine production, distinct plant protection recommendations for these relatively new cultivars are missing. A total of 31 different quantitative trait loci (QTL) conferring resistance to downy mildew have been identified [7–9]. Despite the high number of resistance loci identified, most downy mildew-resistant cultivars grown in Europe rely on resistance conferred by the major dominant loci *Rpv1* (*Resistance to P. viticola 1*)*, Rpv3*, *Rpv10* and *Rpv12*. A successful defense results in the occurrence of necrotic lesions, limited mycelial growth and a reduced number of sporangia in *Rpv12-*, *Rpv10-* and *Rpv3-*cultivars [2, 10–14]. The *Rpv1-* and *Rpv3*-loci originated from native North American grapevine species [12, 15], while *Rpv10-* and *Rpv12*-loci were introgressed from the Asian species *Vitis amurensis* [14, 16]. Resistance genes of the *Rpv1-* and *Rpv3*-loci encode specific receptor proteins with nucleotide-binding domains and leucine-rich-repeats (NB-LRRs), allowing a specific recognition of *P. viticola* at the site of infection [17, 18]. Plants possess a basal immune system, which is based on the recognition of pathogen-associated molecular patterns (PAMPs) or endogenous molecules, which are referred to as damage- or danger-associated molecular patterns (DAMPs) by transmembrane pattern recognition receptors (PRRs) on the external surface of the host cell [19]. This recognition activates PAMP-triggered immunity (PTI), which hinders infection by non-adapted pathogens [20]. In contrast, adapted pathogens deliver effectors, which suppress PTI and enable the pathogen to colonize the host plant (virulent pathogen isolates). Effectors are recognized by specific resistance proteins with NB-LRRs, during an incompatible plant-pathogen interaction [21]. This recognition results in a resistance of the plant to the pathogen and a transcriptional activation of several defense genes (ETI; effector-triggered immunity; avirulent pathogen) [20, 22, 23]. Interestingly, whereas development of ETI has been linked to co-evolution of mildew strains with wild American grapevine species, the co-evolutionary history of Asian wild grapevine species and mildew pathogens is still not completely understood [24–26].

During ETI, signal transduction pathways like mitogen-activated protein (MAP) kinases and WRKY transcription factors are activated, which in turn results into a rapid influx of calcium ions, a burst of reactive oxygen species (ROS), transcription of pathogenesis-related (PR) proteins and biosynthesis of phytoalexins, which finally leads to a hypersensitive response (HR) [22, 27, 28]. Due to evolutionary host adaption of pathogens, cultivation of resistant plants, which rely on a single resistance locus are at risk of breakdown by new virulent isolates of pathogens that can escape detection by the resistance proteins [29, 30]. The arise of *P. viticola* isolates, which are able to overcome resistance mediated by the *Rpv3*-locus is a well-known phenomenon in Europe and was shown in multiple studies [14, 31–33]. To minimize this threat, current grapevine breeding strategies aim to combine several *Rpv*-loci within the one genotype, resulting in new FRC with durable resistance [3, 6, 34]. The durability of resistance of these new grapevine cultivars is of particular importance in viticulture, since grapevines are permanent fruit crops growing for up to 30 years in vineyards [35]. In the recent years the first FRC with polygenic resistance against *P. viticola* have been registered [36–38]. However, detailed knowledge of the mechanisms underlying the resistance conferred by the loci used in these new breeding lines is lacking.

One class of bioactive secondary metabolites, the phytoalexins, are thought to play a crucial role in the defense against *P. viticola* [32, 39, 40]. The phytoalexin *trans*-resveratrol is the precursor for a number of more fungitoxic stilbenes, such as *ε-*viniferin and *trans*-pterostilbene [41, 42]. Besides this, Chang and co-workers proposed that *trans*-resveratrol could also play a role as signaling molecule during HR [43]. Initial investigations into the mechanism of *Rpv12*-mediated resistance showed that *trans*-resveratrol accumulated within 12 h post inoculation (hpi) and a HR was observed between 24 to 48 hpi [14, 39]. For *Rpv10*-mediated resistance an increase in *trans*-resveratrol synthesis was observed as early as 7 hpi and higher levels at 48 hpi in *Rpv10/Rpv3-* cultivars [44]. *Rpv3*-mediated resistance has been shown to be associated with cell death after 32 hpi, an increased level of *trans*-resveratrol between 24 and 72 hpi and the accumulation of fungitoxic stilbenes [32, 45, 46].

In this study the resistance mechanisms mediated by the *Rpv10-*, *Rpv3-* and/or *Rpv12-*loci on downy mildew development, sporulation ability, onset of PCD, production of hydrogen peroxide and *trans*-resveratrol levels were evaluated and compared. Furthermore, a new *P. viticola* isolate was described overcoming the polygenic *Rpv3/Rpv12*-mediated resistance. On-farm experiments demonstrate that the deployment of new FRC containing an *Rpv3-* and/or *Rpv12-*loci can markedly reduce fungicide applications in the vineyard, but also indicate the risk of development of *P. viticola* isolates that are able to overcome plant resistance by omitting plant protection treatments.

## Results

### Evaluation of downy mildew resistance conferred by different *Rpv-*loci

**Fig. 1:**
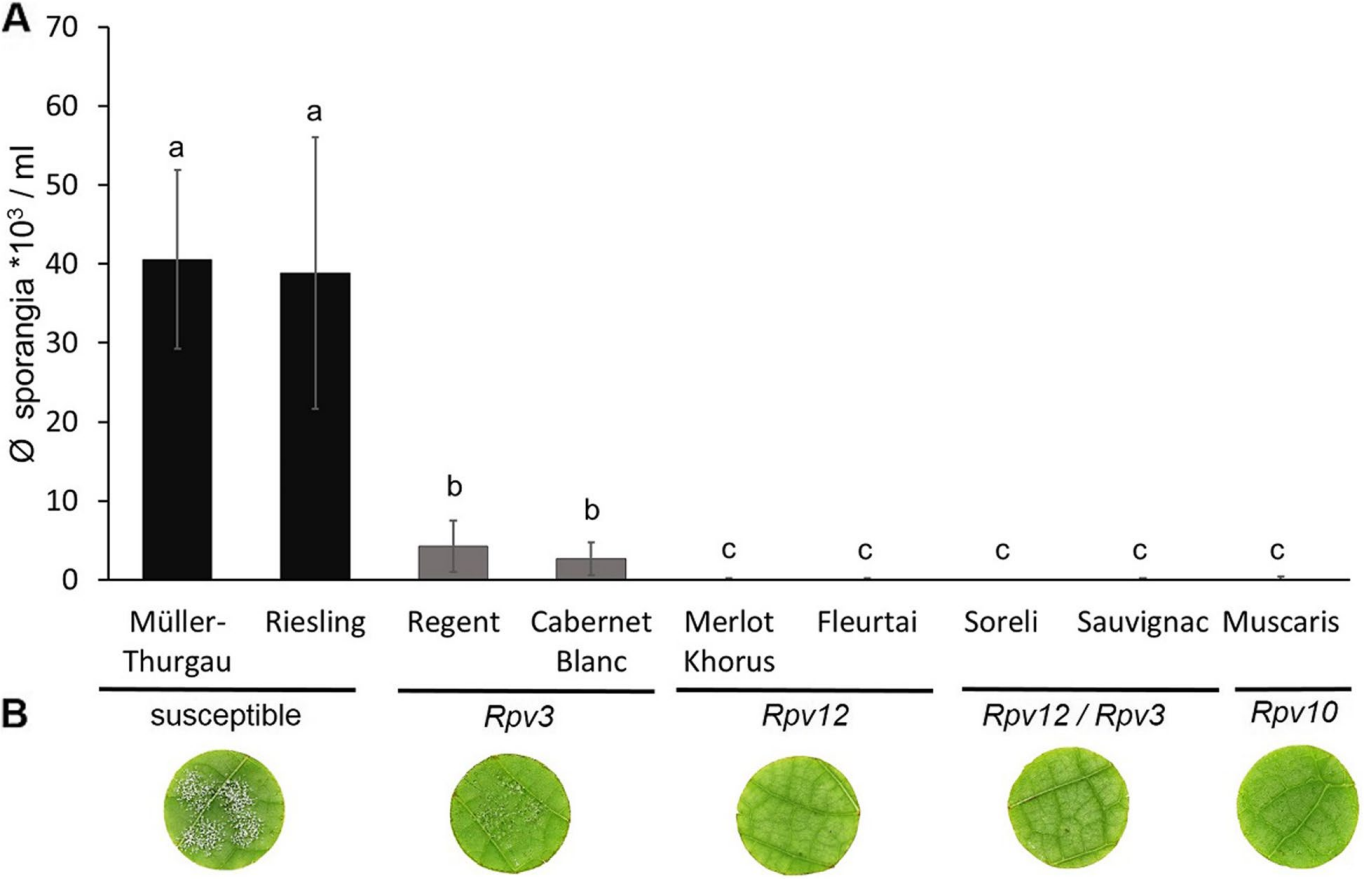
Growth and sporulation of *Plasmopara viticola* isolate *avrRpv*^+^ on susceptible and resistant cultivars. Leaf discs of susceptible cultivars ‘Müller-Thurgau’ and ‘Riesling’, *Rpv3-*cultivars ‘Cabernet Blanc’ and ‘Regent’, *Rpv12*-cultivars ‘Merlot Khorus’ and ‘Fleurtai’, *Rpv12/Rpv3*-cultivars ‘Soreli’ and ‘Sauvignac’ and the *Rpv10*-cultivar ‘Muscaris’ were inoculated with the *avrRpv*^+^*P. viticola* isolate. **A** Evaluation of *P. viticola* sporulation on leaf discs. Counted sporangia after 6 days post inoculation (dpi) were shown. Bars represent the average of three independent experiments (*n* = 63). Error bars show standard deviation. Kruskal-Wallis and Steel-Dwass-Critchlow-Fligner test were used to analyse data and perform multiple pairwise comparison of the effect of *avrRpv*^+^ isolate infection on different cultivars. Means with different letters (**a,b,c**) are significantly different (*p* < 0.05). **B** Representative pictures of leaf discs (susceptible cultivar - ´Riesling´; *Rpv3-*cultivar - ´Cabernet Blanc´; *Rpv12*-cultivar - ´Fleurtai´; *Rpv12/Rpv3*-cultivar - ´Sauvignac´; *Rpv10*-cultivar - ´Muscaris´) were taken at 6 dpi

To compare the level of resistance conferred by different *Rpv*-loci against *P. viticola*, susceptible grapevine cultivars i.e. ‘Müller-Thurgau’ and ‘Riesling’ and grapevine cultivars with different *Rpv*-loci i.e. ‘Cabernet Blanc’ and ‘Regent’ (*Rpv3),* ‘Merlot Khorus’ and ‘Fleurtai’ (*Rpv12*), ‘Soreli’ and ‘Sauvignac’ (*Rpv12/Rpv3)* and ‘Muscaris’ (*Rpv10*) were inoculated with the *P. viticola* (*avrRpv*^+^ isolate). To investigate the possible influence of the background genotype on the resistance response, two different cultivars were analyzed that contained *Rpv12-*, *Rpv3-* or *Rpv12/Rpv3-*loci. The production of sporangia was significantly higher on susceptible genotypes (~ 40,000 sporangia ml^− 1^) than on resistant genotypes (~ 0–4000 sporangia ml^− 1^) at 6 days post inoculation (dpi) (Fig.A; B). However, even though *P. viticola* sporulation was strongly reduced in all resistant cultivars, significant differences in the degree of resistance were observed with the *Rpv3-*cultivars showing sporulation levels of ~ 2500–4000 sporangia ml^− 1^ compared to cultivars, containing *Rpv10-, Rpv12-* or *Rpv12/Rpv3-*loci showing sporulation levels of less than 1000 sporangia ml^− 1^ (Fig. 1A; B).

### Timing of cell death and impairment of mycelial growth is dependent on the type of resistance locus

**Fig. 2:**
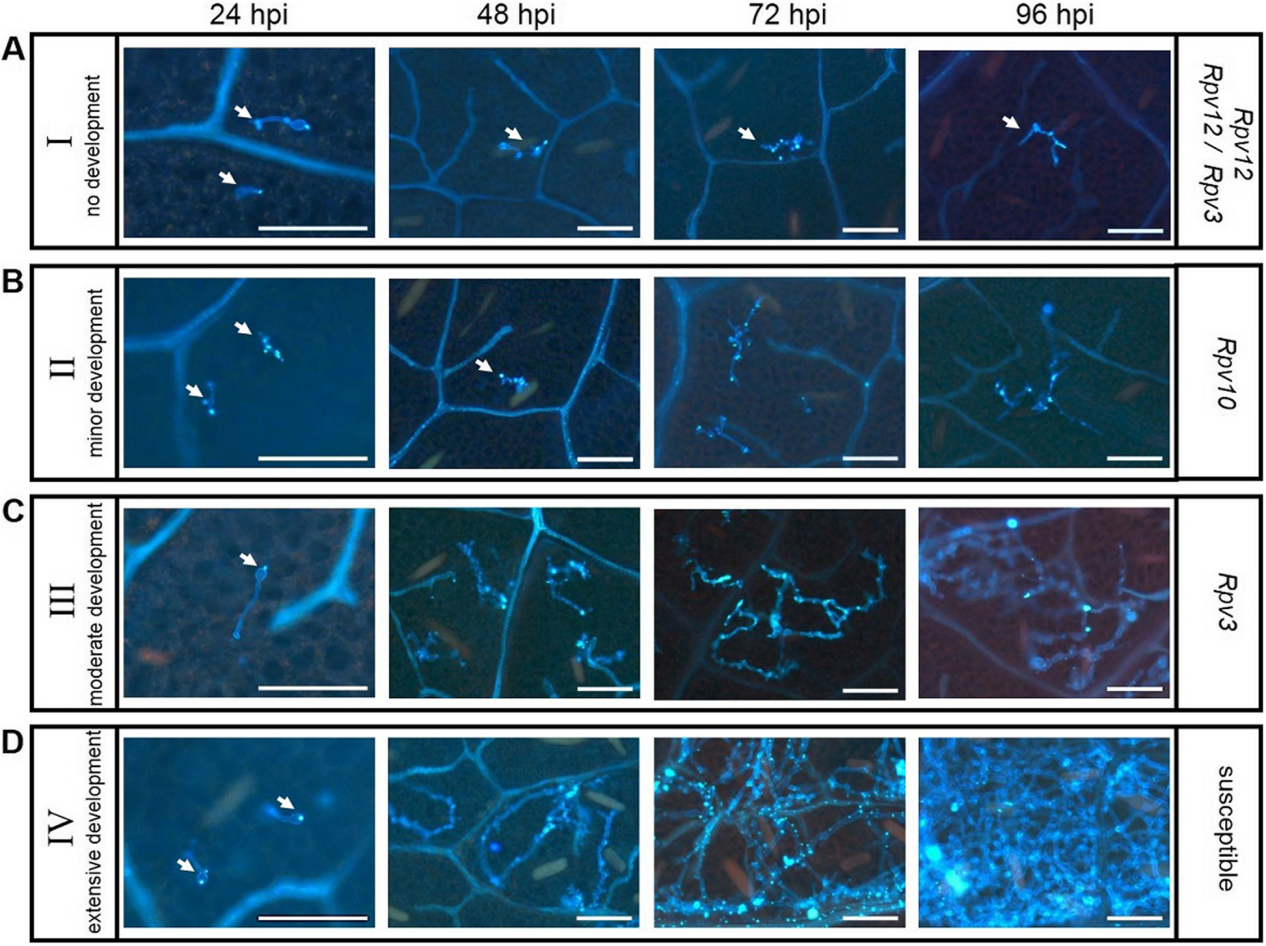
Representative *Plasmopara viticola* development in leaves of susceptible and resistant cultivars. Mycelial growth of the *P. viticola* isolate (*avrRpv*^+^*)* was evaluated using UV epifluorescence after aniline blue staining at 24 hours post inoculation (hpi), 48 hpi, 72 hpi, 96 hpi (left to right). Representative *P. viticola* development from cultivars within each category are shown: **A** category I – no development of *P. viticola* mycelium in *Rpv12-* and *Rpv12/Rpv3-*genotypes (pictures from ´Fleurtai´), **B** category II – minor development of *P. viticola* mycelium in *Rpv10-*genotypes (pictures from ´Muscaris´), **C** category III – moderate development of *P. viticola* mycelium in *Rpv3-*genotypes (pictures from ´Cabernet Blanc´) and **D** category IV – extensive development of *P. viticola* mycelium in susceptible genotypes (pictures from ´Riesling´). Arrows highlight small *P.viticola* infection structures. Images are representative of three biological replicates and three independent experiments. Scale bars correspond to 100 μm

For all histochemical analysis, leaf discs from susceptible and resistant genotypes were inoculated with *P. viticola* isolate *avrRpv*^+^. For aniline blue staining leaf discs were sampled at 24, 48, 72, 96 hpi to observe the development of *P. viticola* mycelium (Fig. 2A-D). Based on the observations the development of *P. viticola* was categorized as follows: category I – no development of *P. viticola* mycelium, category II – minor development, category III – moderate development and category IV – extensive development (Fig. 2A-D). At 24 hpi, no differences in either the ability of zoospores to attach to the stomata or germ tube development or the formation of primary hyphae were observed between the different genotypes (Fig. 2A-D). However, at 48 hpi, mycelial growth was impaired in *Rpv12-*, *Rpv12/Rpv3-* and *Rpv10-*genotypes, whereas in *Rpv3-* and susceptible genotypes the mycelial growth progressed (Fig. 2A-D). Between 48 to 96 hpi, no further mycelium growth was observed in *Rpv12-* and *Rpv12/Rpv3-*genotypes (Fig. 2A) and a small amount of mycelium development was observed for the *Rpv10*-genotype (Fig. 2B). Of the resistant genotypes, the most mycelial development was observed in the *Rpv3-*genotypes (Fig. 2C), but this was still moderate compared to the susceptible genotypes, which were entirely colonized by mycelium at 96 hpi (Fig. 2D).

**Fig. 3:**
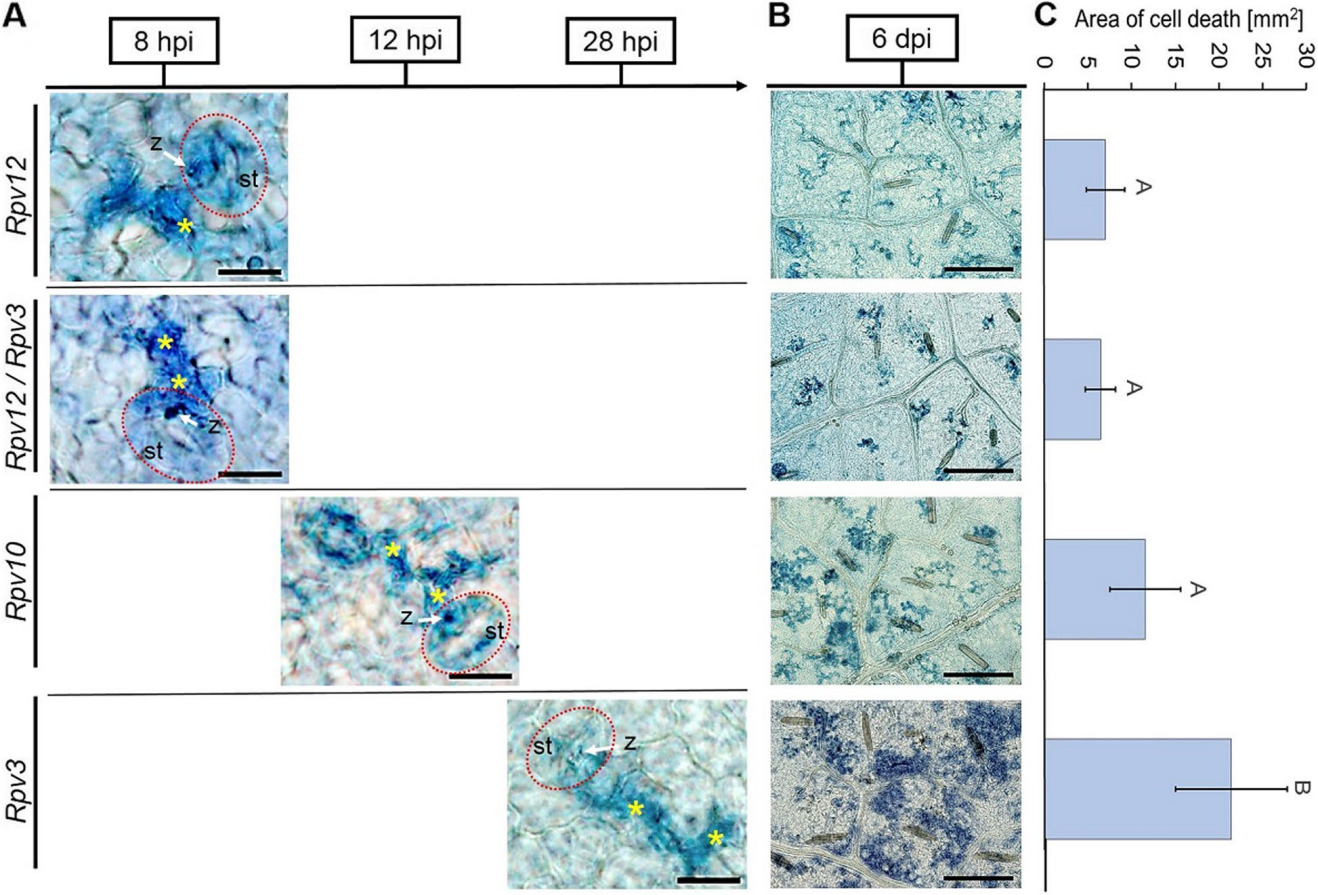
Comparative timing of induction of programmed cell death in *P. viticola*-infected leaves of different resistant genotypes. Leaf discs were infected with *avrRpv*^+^. **A** PCD is first observed after 8 hpi in *Rpv12-* (´Fleurtai´) and *Rpv12/Rpv3-* (´Sauvignac´) genotypes, after 12 hpi in *Rpv10-* (´Muscaris´) and after 28 hpi in *Rpv3-* (´Regent´) genotypes. Yellow asterisks indicate trypan blue stained cells showing cell death, st, stomata outlined with dashed line in red; z, encysted zoospore marked with a white arrow. Scale bar correspond to 20 μm. **B** Area of PCD after 6 dpi. Scale bar correspond to 200 μm. Images are representative of three biological replicates and three independent experiment. **C** Area of cell death in mm^2^ after 6 dpi. Bars represent the average of five independent measurements. Error bars show standard deviation. ANOVA and Tukey’s HSD (Honestly Significant Difference) test were used to compare the cell death area between the different genotypes. Means with different letters (**A,B**) are significantly different (*p* < 0.05)

The time point at which programmed cell death (PCD) was induced during infection was determined by sampling discs at 6, 8, 10, 12, 24, 28 and 32 hpi and staining with trypan blue. Co-staining of zoospores allowed the identification of infected stomata. The first appearance of PCD was observed in leaf cells of the *Rpv12*- and the *Rpv12/Rpv3*-genotypes at 8 hpi, followed by the *Rpv10*-genotype at 12 hpi and the *Rpv3-*genotypes significantly later at 28 hpi (Fig. 3A). No PCD was observed at any time points in the infected susceptible cultivar or the mock inoculation (water) control (Additional file 1A). After 6 days, the infected susceptible cultivar shows cell death, which is presumably caused by nutrient deprivation of the pathogen and therefore does not represent a defense reaction (Additional file 1B). To quantify the differences in the area of PCD, trypan blue staining was analyzed at 6 days post inoculation (dpi) in *Rpv12*-, *Rpv12/Rpv3*-, *Rpv10*- and *Rpv3-*genotypes (Fig. 3B;C). The cell death areas were measured using the ROI (region of interest) manager of the Fiji software. Five images of different leaf discs were taken at 20x magnification. Each cell death area was outlined by hand and each ROI was calculated by the program. The mean value in mm^2^ is displayed. The average PCD area at 6 dpi for *Rpv12*- and *Rpv12/Rpv3*-genotypes was found to be ~ 6–7 mm^2^, which was slightly lower, but not statistically significantly different to the *Rpv10-*genotype (~ 11.5 mm^2^). However, the area of PCD observed in *Rpv3-*genotypes was significantly larger compared to the other resistant genotypes and was four times larger than that, observed in genotypes containing the *Rpv12-*locus.

**Fig. 4:**
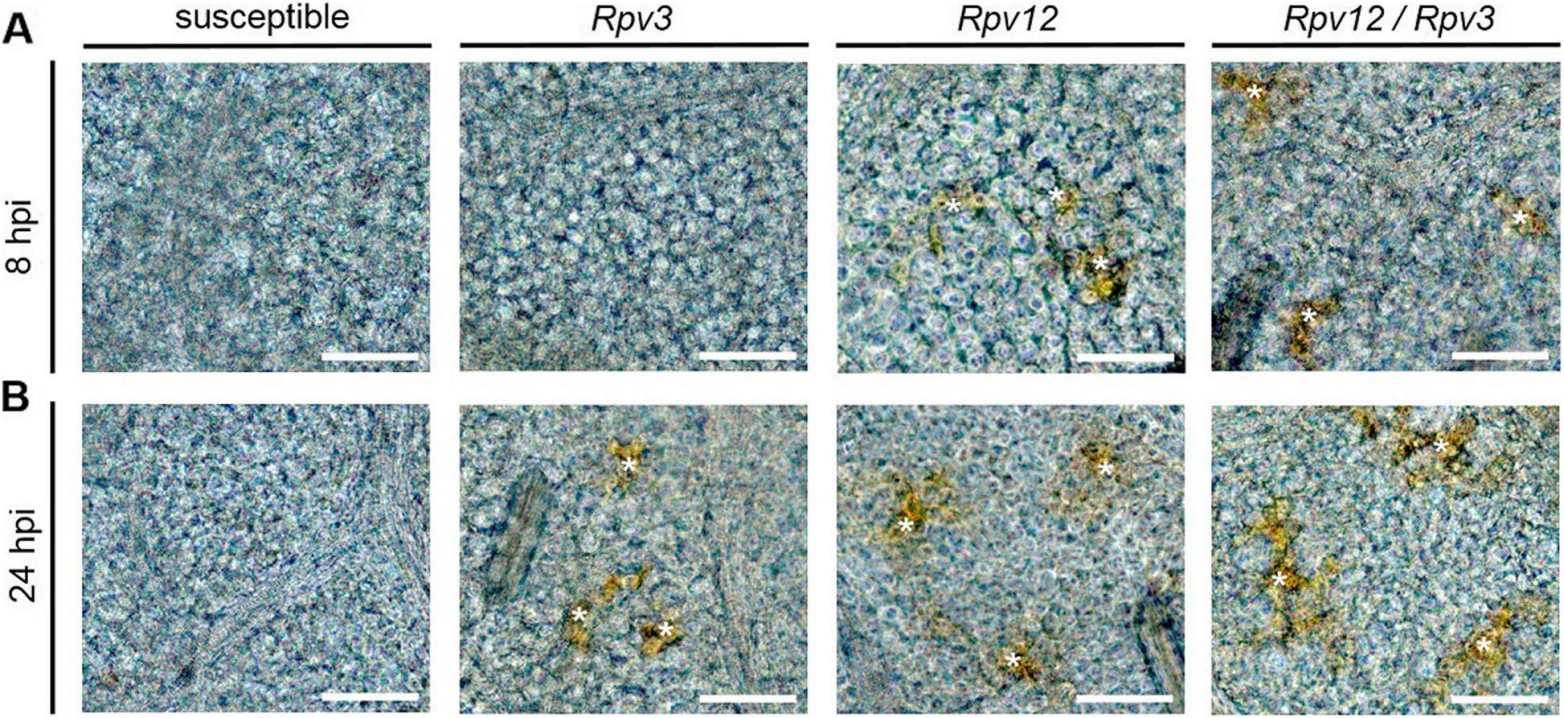
Hydrogen peroxide production at *Plasmopara viticola* infection sites. *P. viticola (avrRpv*^+^*)* was used to inoculate leaf discs of susceptible genotype (´Müller-Thurgau´) and resistant *Rpv3-* (´Regent´), *Rpv12-* (´Fleurtai´) and *Rpv12/Rpv3-*genotype (´Sauvignac´). Samples were taken at 0, 2, 4, 6, 8, 10, 12, 24, 28, 32 and 36 hpi. Asterisks mark 3, 3′-diaminobenzidine-tetrahydrochloride stained cells indicating hydrogen peroxide formation. Images are representative of three biological replicates in three independent experiments (*n* = 9). Scale bars correspond to 50 μm

As the *Rpv12*- and *Rpv12/Rpv3*-genotypes showed the earliest onset of PCD and the *Rpv3-*genotypes the latest onset, as well as the greatest difference in mycelium development (Fig. 2A-D), these genotypes were analyzed for the presence of an oxidative burst associated with PCD. Diaminobenzidine (DAB) staining confirmed hydrogen peroxide (H_2_O_2_) production coincided with the induction of PCD at infection sites in all resistant genotypes. Hydrogen peroxide was detected in *Rpv12-* and *Rpv12/Rpv3*-genotypes at 8 hpi but not until 24 hpi in *Rpv3-*genotypes (Fig. 4A; B). No hydrogen peroxide formation was observed in the susceptible genotype (Fig. 4A; B) or mock inoculation (water) controls (Additional file 2B).

### *Trans*-resveratrol production is more highly induced in response to *P. viticola* and precedes PCD

**Fig. 5:**
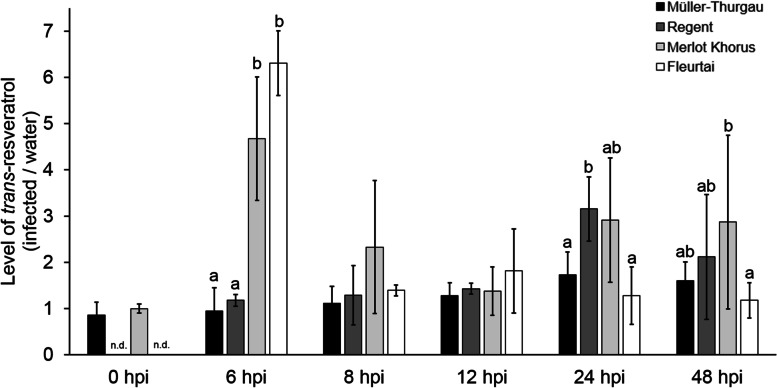
Level of *trans-*resveratrol (infected/water control) in susceptible, *Rpv3-* and *Rpv12-*genotypes in response to *P. viticola* (*avrRpv*^+^) inoculation. *Trans-*resveratrol was measured in leaf discs after inoculation with *avrRpv*^*+*^ isolate or mock inoculated (water) control of the following genotypes: ´Müller-Thurgau´ (susceptible), ´Regent´ (*Rpv3*)*,* ´Merlot Khorus´ and ´Fleurtai´ (*Rpv12)*. Samples were collected at 0, 6, 8, 12, 24 and 48 hpi. Bars represent the average of three or two independent measurements (*n* = 3, ´Fleurtai´ *n* = 2) of 10 pooled biological replicates [ng g^− 1^ fresh weight] normalized against water control. Error bars show standard deviation. Kruskal-Wallis and Steel-Dwass-Critchlow-Fligner test was used for multiple pairwise comparison of the effect of the inoculation with *avrRpv*^*+*^ on different cultivars. Statistical analysis compares the significance of all samples at one time point, different letters (**a, b**) are significantly different (*p* < 0.05); not detected (n.d.). Results of three independent experiments are shown

A previous study proposed that *trans-*resveratrol may have a role as a signaling molecule for the induction of PCD, in addition to its known role as a phytoalexin [47]. We measured *trans-*resveratrol biosynthesis over 48 hpi with *P. viticola* in susceptible and resistant genotypes in three independent experiments, except for the genotype ´Fleurtai´, which was only analysed in two experiments. Figure 5 shows that *trans*-resveratrol levels in *Rpv12*-genotypes were highly elevated in leaf discs 6 hpi with downy mildew in comparison to mock inoculated (water) leaf discs. In contrast, there was little or no induction of *trans*-resveratrol in the *Rpv3-* or susceptible genotypes. After 6 hpi the *trans*-resveratrol level in *Rpv12*-genotypes decreased such that there was no significant difference between any of the genotypes at 8 and 12 hpi. For the *Rpv3-*genotype an increase of *trans-*resveratrol was then observed from 12 to 24 hpi resulting in a significant higher level at 24 hpi compared to the susceptible genotype.

### *Rpv12/Rpv3* confers a higher degree of resistance in the vineyard than *Rpv3-*cultivar under strong infection pressure

**Fig. 6:**
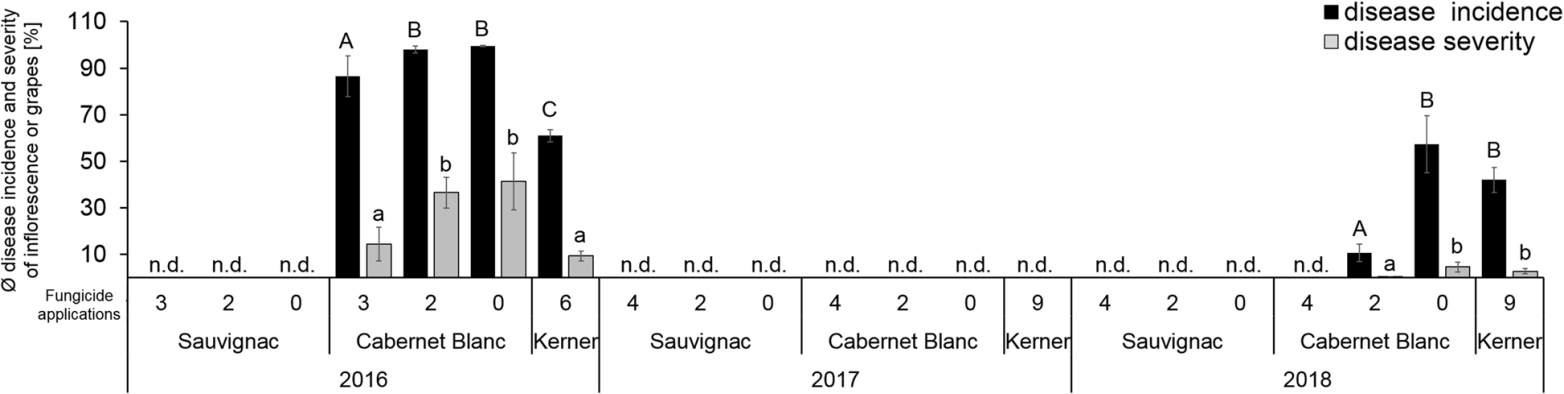
*Plasmopara viticola* disease incidence and severity in inflorescences (BBCH 71 – 2016) and grapes (BBCH 75 and 88 – 2017, 2018). Disease incidence (black bars) and disease severity (grey bars) were quantified over three seasons in the grapevine cultivars ´Sauvignac´ (*Rpv12/Rpv3*), ´Cabernet Blanc´ (*Rpv3*) and ´Kerner´ (susceptible), differing in the number of fungicide applications during the season. The bars show mean values for two different locations in 2016 (n (grapes/application number) = 200) and from three different locations in 2017 and 2018 (n (grapes/application number) = 300) for ´Sauvignac´ and ´Cabernet Blanc´. Data from ´Kerner´ are mean values of one location *n* = 100. Error bars show standard error (SEM); no infections detected (n.d.). Kruskal-Wallis and Conover-Iman test was used to compare the disease incidence (**A, B, C**) and severity (**a, b, c**) for each year, *p* < 0.05

On-farm experiments were carried out in three commercial vineyards in Rhine-Palatinate region with varying in the number of fungicide treatments (Additional file 3), to evaluate the potential benefit of FRC in reducing the need for fungicide application. The recommended spray program for this region for susceptible cultivars is 9–11 treatments per season, depending on the infection pressure [48, 49]. However, there is no information available as to the recommended fungicide spray program for FRCs. Therefore, we undertook experiments using three different fungicide treatment programs (0, 2 or 4 applications per season) (Additional file 3), applied to the FRCs `Sauvignac` (*Rpv12/Rpv3*) and `Cabernet Blanc` (*Rpv3*). It was not possible to apply these reduced fungicide spray programs on susceptible cultivars, due to the high probability of complete crop loss for the grape growers. Therefore, the results of our reduced fungicide spray programs on *P. viticola* disease incidence and severity on the FRCs was compared with disease incidence and severity observed on the susceptible cultivar `Kerner`, which was treated as per normal commercial practices (2016: 6 applications until time of evaluation; 2017 and 2018: 9 applications) (Fig. 6). *Plasmopara viticola* disease incidence and severity on inflorescences, grapes and leaves were assessed weekly over three growing seasons.

No *P. viticola* infection was detected on ‘Sauvignac’ (*Rpv12/Rpv3)* over the 3 years of the trial for any of the spray variants in the three different vineyards, which translates to a fungicide saving of 100%. In contrast, *Rpv3-*cultivar ‘Cabernet Blanc’ showed a higher susceptibility to *P. viticola* in 2016 and 2018 seasons (Fig. 6). During the 2016 season, disease incidence and severity on inflorescences on ‘Cabernet Blanc’ after three treatments were significant lower compared to 2 and 0 treatments in grapes (Fig. 6) and leaves (Additional file 4). Despite the high disease incidence of ~ 80% the disease severity of ~ 14% was low enough to achieve yield and quality comparable to the susceptible cultivar `Kerner`, which showed similar disease incidence and severity levels, while treated 6 times at monitoring (Fig. 6). This represents a 50% reduction in fungicide applications for ‘Cabernet Blanc’ compared to `Kerner` until BBCH 71 (Fig. 6). In 2016, *P. viticola* infection pressure was extremely high, therefore the study was stopped at BBCH 71 (start of fruit development) and fungicide application was performed as per normal commercial practices to ensure plant health.

In 2017 the weather conditions were not sufficient to trigger a primary infection or sporulation in the susceptible, *Rpv3-* and *Rpv12/Rpv3*-cultivars, resulting in 100% fungicide saving. Disease incidence and severity of *P. viticola* was lower in 2018, compared to 2016, but again showed that unsprayed grapevines displayed a significantly higher disease incidence and severity on ‘Cabernet Blanc’ grapes (Fig. 6) and leaves (Additional file 4). In 2018 the susceptible cultivar was treated 9 times resulting in comparable disease incidence and severity as found in the untreated ‘Cabernet Blanc’ grapes. Four fungicide applications gave complete control of *P. viticola* infection on ‘Cabernet Blanc’ grapes (55% reduction of fungicide applications) and two treatments were sufficient to result in no yield and quality loss (75% fungicide application reduction) (Fig. 6).

### Identification of a new *Plasmopara viticola* isolate that can overcome both *Rpv12-* and *Rpv3*-mediated resistance

Mutations driven by selection processes are the main cause for adaptation of pathogens to their hosts. We previously reported on the identification of an *avrRpv3*^*−*^ isolate that is virulent on ‘Cabernet Blanc’ (*Rpv3*) from one of the vineyards in the Rhineland-Palatinate region that was used in this fungicide study [32]. More recently, we observed downy mildew growing on the cultivar ‘Sauvignac` (*Rpv12/Rpv3*) in an isolated vineyard of Valentine Blattner (VB) in Soyhières, Switzerland (2019). This VB-isolate was collected and propagated and tested on a range of different grapevine genotypes*.* To evaluate whether the VB-isolate has resistance-breaking properties the amount of newly produced sporangia, mycelium development and induction of cell death were investigated by inoculating a range of susceptible and downy mildew-resistant cultivars containing different *Rpv*-loci.

**Fig. 7:**
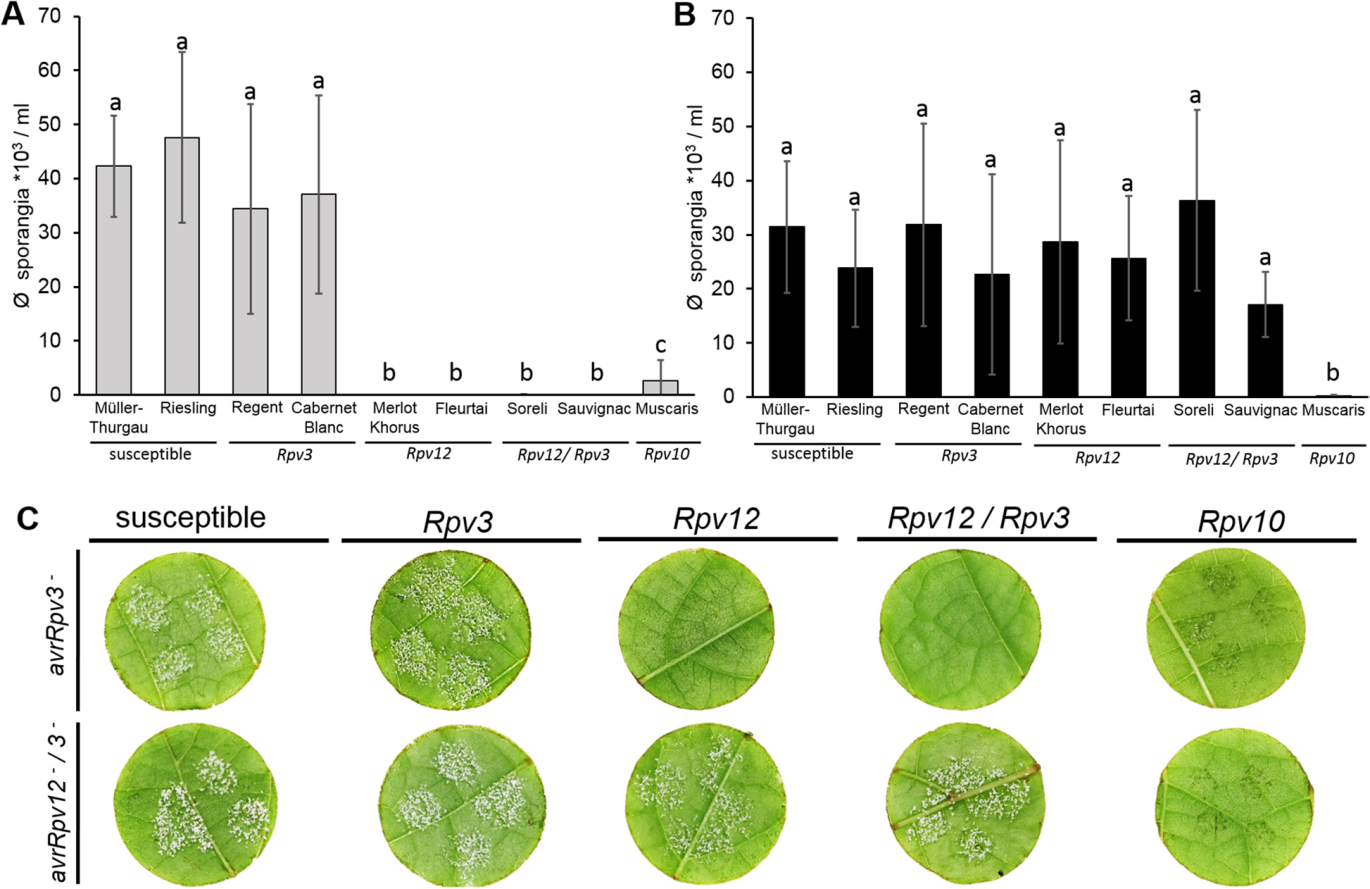
Growth and sporulation of *avrRpv3*^−^ and *avrRpv12*^*−*^*/3*^*−*^*Plasmopara viticola* isolates on susceptible and resistant cultivars. Leaf discs of susceptible cultivars ‘Müller-Thurgau’ and ‘Riesling’, *Rpv3-*cultivars ‘Cabernet blanc’ and ‘Regent’, *Rpv12*-cultivars ‘Merlot Khorus’ and ‘Fleurtai’, *Rpv12/Rpv3*-cultivars ‘Soreli’ and ‘Sauvignac’ and the *Rpv10*-cultivar ‘Muscaris’ were inoculated with *P. viticola* isolates. Quantitative evaluation of sporulation of *P. viticola* isolates (**A**) *avrRpv3*^*−*^ isolate and (**B**) VB (*avrRpv12*^*−*^*/3*^*−*^) isolate on leaf discs. Sporangia were counted 6 days post inoculation (dpi). Bars represent the average of three independent experiments (*n* = 63). Error bars show standard deviation. Kruskal-Wallis and Steel-Dwass-Critchlow-Fligner test was used for multiple pairwise comparison of the effect of the two different isolates on different cultivars. Means with the different letters (**a, b, c**) show significant differences for infection (*p* < 0.05). **C** Pictures of representative leaf discs of susceptible cultivar - ´Riesling´; *Rpv3-*cultivar - ´Cabernet Blanc´; *Rpv12*-cultivar - ´Fleurtai´; *Rpv12/Rpv3*-cultivar - ´Soreli´; *Rpv10*-cultivar - ´Muscaris´ were taken at 6 dpi with the *avrRpv3*^*−*^ (top) and *avrRpv12*^*−*^*/3*^*−*^ (bottom) isolate

The level of sporulation of the *avrRpv3*^*−*^ isolate was significantly higher on all susceptible and *Rpv3–1* cultivars ~ 34,000–47,000 sporangia ml^− 1^) compared to *Rpv12*- (~ 0–60 sporangia ml^− 1^) and *Rpv10*- (~ 2600 sporangia ml^− 1^) cultivars (Fig. 7A). However, no significant difference in sporulation was detected between *Rpv12-*, *Rpv3-*, *Rpv12/Rpv3-* and susceptible cultivars (~ 17,000–36,000 sporangia ml^− 1^) inoculated with the VB-isolate showing its ability to overcome *Rpv3*- and *Rpv12*-mediated grapevine resistance (*avrRpv12*^*−*^*/3*^*−*^)*.* However, the VB-isolate was not able to overcome *Rpv10*-mediated resistance, as the *Rpv10*-cultivar showed a significant and strongly reduced number of sporangia (less than 200 sporangia ml^− 1^) (Fig. 7A). These results demonstrate for the first time the development of a *P. viticola* isolate, which is able to simultaneously overcome both the *Rpv3-* and *Rpv12*-loci.

To further investigate the effects of the resistance breaking isolates on pathogen development and PCD, aniline blue and trypan blue staining were performed. Once a pathogen overcomes resistance, mycelial development was comparable to that in susceptible cultivars and no cell death was observed. (Figs. 2 and 3, Additional files 5 and 6). The results confirm the resistance-breaking properties described in the sporulation assay (Fig. 7).

## Discussion

### Differences in the defense responses mediated by the *Rpv3-, Rpv12-* and *Rpv10-* loci in response to *P. viticola* infection

In plant-pathogen interactions with biotrophic pathogens, the initiation of PCD is associated with plant resistance and can be visible as macro or micro-necrotic lesions at the infection site [50]. Macroscopically visible lesions were reported in multiple resistant grapevine genotypes within 2–10 dpi but these studies reported no macroscopic difference in type and timing of the plant defense response in *Rpv3-* and *Rpv12-*locus containing genotypes [12, 14, 44, 51]. In contrast, this study focused on the early response to *P. viticola* between 6 and 32 hpi and investigated the different genotypes under the same experimental conditions and with the same *P. viticola* isolates. The comparison of the initiation and development of grapevine defense responses against *P. viticola* infection in resistant cultivars containing different downy mildew resistance loci revealed clear temporal differences in the onset of PCD in response to *P. viticola* in *Rpv12*, *Rpv10*- and *Rpv3-*genotypes (Fig. 3A). An early onset of PCD was observed in *Rpv12*- (8 hpi) and *Rpv10-*genotypes (12 hpi), but a much slower response was observed in *Rpv3-*genotypes (28 hpi) (Fig. 3A). The rapid induction of PCD within 8 hpi in *Rpv12*-genotypes (Fig. 3A), results in the early arrest of pathogen growth with no mycelial development visible (Fig. 2A). In comparison, *Rpv10*-mediated PCD was induced approximately 4 hours later (Fig. 3A), resulting in a small amount of mycelial development compared to the *Rpv12-*genotypes (Fig. 2B). In *Rpv3-*genotypes, PCD was not observed in our experiments until 28 hpi (Fig. 3A), allowing moderate development of the pathogen (Fig. 2C). These results confirm the observations reported by Eisenmann et al. [32], who found PCD was not induced in the *Rpv-3* genotype at 32 hpi. These results are in good accordance with the theory that the timing of the defense initiation define the resistance level and successful plant defense [52–54].

The first interaction of *P. viticola* with the plant cell takes place after the development of the pathogen haustoria, which are the cell-wall-penetrating and feeding structures of *P. viticola.* They are established and invaginate the plasma membrane of the parenchyma cells between 3.5 and 6 hpi [55, 56]. The fast and local PCD observed in *Rpv12*-genotypes at 8 hpi and the complete growth inhibition of the pathogen stands in contrast to the late cell death in *Rpv3-*genotypes observed at 28 hpi and the ongoing growth of the pathogen after first haustorium establishment. This early and late timing of PCD induction correlates with a differences in the area of PCD after 6 days post inoculation (Fig. 3A-C) presumably caused by the ongoing development of new haustoria for *Rpv3*-genotypes, resulting in trailing PCD. The late cell death observed in *Rpv3*-genotypes after 28 hpi is presumably not caused due to nutrient deprivation by the pathogen, since cell death reaction was not observed in the susceptible cultivar at 32 hpi (Additional file 1A). One can only speculate at this stage as to the reason(s) for the observed differences in timing of the induction of PCD mediated by the different *Rpv* loci. One possible factor may be differences in the timing of secretion of the *P. viticola* effectors that are recognized by the NB-LRR resistance protein encoded by the *Rpv* locus or the expression of defense related genes. For example, Yin et al. [57], showed that the recognition of the effectors secreted during *P. viticola* infection show very different transcriptional profiles with some expressed highly within the first 12 hpi and others not induced until 24 hpi [57]. Other differences may also relate to recognition of the activated NB-LRR protein by the downstream signaling components.

### Role of reactive oxygen species and stilbenes in the defense response to downy mildew

Effector-triggered immunity (ETI) is not only characterized by the induction of PCD but also a number of other cellular events including the rapid production of reactive oxygen species (ROS) and the release of antimicrobial compounds, such as phytoalexins, which are involved in successful limitation of pathogen development [22, 40, 58]. Furthermore, ROS like hydrogen peroxide have been proposed to act as signaling molecules for activation of defense genes and the HR [59, 60].

To obtain further insights into the observed timing differences mediated by different downy mildew resistance loci, the accumulation of hydrogen peroxide and the phytoalexin *trans*-resveratrol were compared in genotypes containing the *Rpv12-*locus with genotypes only containing *Rpv3-*locus*.* Our results demonstrate that hydrogen peroxide was produced within 8 hpi at the infection site in *Rpv12*-genotypes which co-incided with the appearance of PCD (Fig. 4). In contrast, hydrogen peroxide was not detected in *Rpv3-*genotypes until 24 hpi (Fig. 4), which was shortly before PCD and was first observed in leaf cells of this genotype (Fig. 3).

*Trans*-resveratrol has also been proposed as signal molecule for PCD [43]. Chitarrini et al. [39] evaluated *trans*-resveratrol accumulation in *Rpv12*-genotypes but their first sampling point was at 12 hpi, which is well after the first appearance of PCD in *Rpv12-*genotypes. Our results show a significant increase of the level of *trans*-resveratrol at 6 hpi, which is prior to the observed production of ROS and the appearance of PCD at 8 hpi (Figs. 3 and 4A). As the accumulation of *trans*-resveratrol precedes the first occurrence of ROS and PCD in *Rpv12*-genotypes, it is tempting to speculate that *trans*-resveratrol, or another stilbene derived from *trans*-resveratrol, may act as an inducer of ROS production and PCD. Indeed, metabolomic and transcriptomic data suggests that the *Rpv12*-mediated resistance relies on a rapid activation of a broad set of inducible responses that take place within 12 hpi [39, 43]. Recently Eisenmann et al. [32] also described elevated *trans*-resveratrol and *ε-*viniferin levels during *Rpv3*-mediated defense response shortly before the onset of PCD at 32 hpi. Our results confirm that observation, with no significant increase of *trans*-resveratrol production in the *Rpv3*-genotype observed until 24 hpi (Fig. 5). Besides the possible role of *trans*-resveratrol as a signaling molecule, it has also been proposed to play a role in the *Rpv3*-mediated defense [32, 45], by acting as precursor for synthesis of the fungi-toxic stilbenes *ε-*viniferin and *trans*-pterostilbene, which suppress the growth and development of *P. viticola* [42]*.*

Interestingly, Chitarrini et al. [39] found no evidence of an increase in *ε-*viniferin levels during *Rpv12*-mediated defense, leading them to conclude that the primary role of *trans*-resveratrol in the *Rpv12*-mediated resistance is as a signaling molecule in ROS formation and initiation of PCD rather than as a precursor of toxic stilbenes.

While the results presented here do not provide any further insights into the role of *Rpv-*mediated stilbene production in suppressing *P. viticola* growth directly, they do provide support the hypothesis that *trans*-resveratrol may act as a signaling molecule for ROS induction and PCD in *Rpv12*- and *Rpv3*-mediated resistance. In the case of the Asian grapevine species and the respective resistance loci *Rpv10* and *Rpv12* it is not clear whether coevolution with mildew pathogens has taken place due to the lack of historical reports [24–26]. Therefore, it cannot be excluded that in contrast to a specific ETI found in American grapevines, another form of immunity (ETI-like immunity) plays a role in the defense reaction of Asian species [24].

### Relative resistance level mediated by the *Rpv3–1*-, *Rpv10*- and *Rpv12*-loci

The resistance conferred by the *Rpv3*-, *Rpv10*- and *Rpv12*-loci to *P. viticola* have been reported in a number of previous studies [12, 14, 51, 61–63]. However, the results of these studies, in terms of the level of resistance mediated by the respective loci, are somewhat contradictory.

For example, Possamai et al. [51] reported that the *Rpv10*-mediated resistance was weaker than that conferred by *Rpv12* and *Rpv3*. Bove and Rossi [61], on the other hand, found comparable levels of downy mildew resistance in *Rpv3-*, *Rpv10-* and *Rpv12-*cultivars. The results of this current study demonstrate clear differences in the level of resistance conferred by the *Rpv3* relative to *Rpv12* or *Rpv10*. Sporulation on *Rpv3-*cultivars was significantly higher compared to *Rpv10-, Rpv12-*cultivars (Fig. 1). These findings are also in line with previous studies showing a higher resistance level of *Rpv10*- and *Rpv12*- compared to *Rpv3-*mediated resistance (Fig. 1) [14, 63, 64]. One possible explanation for these conflicting results could be the different methods used to evaluate levels of resistance. In the present study, resistance was assessed using the measurement of sporulation on individual leaf discs whereas Possamai et al. [51] used a visual scoring system based on the Office International Organisation of Vine and Wine (OIV) [65] scale and Bove and Rossi [61] determined the amount of sporangia of specific lesions. In all studies, the time of the experiments was between April and August and should therefore not be considered as a determining factor for the different results. Another possible explanation for the observed differences in resistance levels between these different studies may be related to plant material sampled for each study. Possamai et al. [51] and Bove and Rossi [61] used leaf discs that were cut from field plants whereas Venuti et al. [14] and this study used leaf discs sampled from greenhouse plants.

### Emergence of a new field isolate that overcomes both *Rpv12-* and *Rpv3–1-*mediated resistance

The emergence of resistance-breaking pathogens by mutations or deletions of avirulence genes and their selection by plant resistance genes have been previously described for several resistant crops such as potato and rice [29, 30, 66, 67]. In addition, oomycetes are known to quickly overcome genetic resistance, as shown for sunflower downy mildew [68] and potato late blight [69]. *Plasmopara viticola* isolates that are able to overcome *Rpv3-*mediated resistance have been described in previous studies, demonstrating that the durability conferred by a single resistance locus may be limited [13, 31–33]. Our results show that an *avrRpv3*^*−*^ isolate has further mutated to also be able to overcome *Rpv12*-mediated resistance (Fig. 7). Therefore, a combination of several resistance loci is of great importance for grapevine breeding [3, 6, 34, 36]. Despite this, our results demonstrate that *P. viticola* isolates overcoming two resistance loci can evolve.

A *P. viticola* isolate overcoming *Rpv3*- and *Rpv12*-loci was detected in a vineyard in which inadequate phytosanitary treatments were performed over the last 3 decades. In this vineyard, an *Rpv3*-cultivar was first planted allowing the selection of an *Rpv3*-breaking isolate. Thereafter an *Rpv12*-cultivar was planted, resulting in the further development of this isolate (Fig. 7A; Additional file 5), designated as *avrRpv12*^*−*^*/3*^*−*^. This shows that if cultivars with the individual resistance loci are grown at the same location with inadequate or no phytosanitary treatments, resistance based on the pyramidization of separate resistance loci can be overcome. These findings demonstrate that the mutated avirulence proteins (*avrRpv3 *and* avrRpv12*) of this isolate are not recognized by the corresponding resistance gene (*R*-gene) product of the *Rpv3-* and *Rpv12-*loci [20]. In contrast, the resistance mediated by the *Rpv10*-locus was not overcome by the *avrRpv12*^*−*^*/3*^*−*^ isolate (Fig. 7). Our results clearly indicate that the *Rpv12-, Rpv10-* and *Rpv3*-mediated resistances are based on three different *R*-gene products detecting different *Avr* proteins of downy mildew. Pyramidization of all three *Rpv-*loci within the same genotype could further increase the durability of new resistant grapevine cultivars.

### The potential of resistant grapevine cultivars to reduce the number of fungicide applications depends on the interplay of genetically based resistance levels, developmental and climatic factors

Data from the Statistical Office of the European Union show that more than 70% of fungicides used in Europe are applied in viticulture [4]. This makes the issue of reducing fungicide applications more significant than for any other agricultural commodity. Fungus-resistant cultivars are one important component of achieving the goal of significantly reducing the use of plant protection products, to reduce the cost of production and the negative impacts of fungicides on the environment and the surrounding population. However, FRCs are currently grown on limited acreages, because of the low acceptance of new cultivars by wine growers and wine consumers. A survey among winegrowers in Germany highlighted the limited knowledge of adjusted plant protection recommendations for FRCs. This survey revealed that FRCs were either treated with the same number of fungicide treatments as susceptible cultivars or, conversely, plant protection was completely omitted [70]. Our study set out to evaluate the performance of FRCs fungicide application programs, which represented a 50–100% reduction in the number of applications required for acceptable disease control on existing susceptible cultivars in the same vineyard. The results showed the complete absence of *P. viticola* infections for the *Rpv12/Rpv3*-cultivar across all treatments and experimental years (Fig. 6, Additional file 1). In contrast, downy mildew infections were observed on the *Rpv3-*cultivar in 2 out of 3 years with the reduced spray treatments (Fig. 6, Additional file 4). However, the level of downy mildew infection observed in those 2 years on the *Rpv3*-cultivar was similar to that observed with the susceptible control cultivar but with 50–100% fewer fungicide applications.

The performance of these *Rpv*-loci in the vineyard, mirrors the results from laboratory-based leaf disc assays (Fig. 1) and highlights the fact that the identity of the resistance locus, present in a FRC has to be taken into consideration when designing the fungicide spray program that will maximize savings on fungicide application but still maintain yield and.

Furthermore, the results suggest that in addition to the genetically determined resistance levels, other factors such as climatic conditions during flowering and fruit set also play a role in the absolute resistance of a cultivar and thus, determine the amount of fungicide treatments required. The flower and fruit development phases are highly susceptible to pathogens [71]. Observations from Kennelly et al. [72] confirm our results observed at BBCH 71 in 2016, which indicated a positive correlation of an extended flowering period with a higher disease severity of inflorescences and berries. In contrast, 2018 the first *P. viticola* infection in the *Rpv3-*cultivar was detected after fruit set in BBCH 75 and did not develop into a severe infection (Fig. 6). The result of the maximum saving found in this study with the *Rpv3-*genotype is in good agreement with described experiences of winegrowers and other [70, 73, 74]. However, these reports did not evaluate the saving potential during high infection periods, as in 2016, where reduction of fungicide applications by ≥75% cannot be compensated by *Rpv3-*cultivars without negative effects on yield or quality.

Our results demonstrate that the omission of plant protection can lead to significant amounts of infection, in some years, in FRCs that rely on *Rpv3*-mediated resistance alone. In two out of three experimental years the omission of plant protection treatments resulted in significantly higher infections with downy (Fig. 6; Additional file 4) and powdery mildew (data not shown). This is in contrast to the findings of Casanova-Gascón et al. [75], who observed no negative effects caused by the omission of plant protection for FRCs, possibly because of the lower infection pressure in their trails. Data over the three trial years showed that no downy mildew disease was observed on the *Rpv12/Rpv3*-cultivar even in the absence of any fungicide applications. However, in a long term the omission of plant protection applications is not a recommendation for practice, because as shown in this study, resistance-breaking isolates can develop if the levels of inoculum in the vineyard are allowed to increase. Taken together, this study highlights that the number of fungicide treatments required is not only defined by the genetically determined resistance levels, but also by other factors such as climatic conditions.

## Conclusion

The experiments revealed an early and locally precise defense response in *Rpv10-*, *Rpv12-* and *Rpv12/Rpv3*-cultivars, whereas a delayed defense response in *Rpv3-*cultivars was observed*.* The differences in the timing of the defense response resulted in different resistance degrees of the various resistant grapevine cultivars, which were verified in lab experiments and on-farm experiments. The use of FRCs in combination with reduced plant protection management strategies offers the possibilities to significantly reduce the amount of fungicides required for grape production. Results obtained from the on-farm studies demonstrated that the deployment of FRCs may save 50–100% of fungicide applications in viticulture. However, the omission of all plant protection applications can ultimately lead to negative effects on yield, quality and even resistance durability. Future studies are needed to evaluate the consequences of reductions of fungicide applications on the emergence of other fungal diseases, such as black rot or *Phomopsis viticola* cane and leaf spot.

## Methods

### Plant material, *Plasmopara viticola* isolates

*Vitis vinifera* cv. ´Müller-Thurgau´, ´Riesling´(susceptible), ´Regent´ (*Rpv3–1*) [11, 34], ´Cabernet Blanc´ (*Rpv3–1*) [32], ´Muscaris´ (*Rpv10*) and ´Sauvignac´ (*Rpv12/ Rpv3*) [38], were regenerated from canes obtained from vineyards of the State Education and Research Center of Viticulture, Horticulture and Rural Development, Neustadt/Weinstr. Germany as described previously [76]. ‘Merlot Khorus’ (*Rpv12*) [37, 61] and ´Soreli´(*Rpv12/ Rpv3)* [37] were regenerated from canes obtained from vineyards in Ungstein, Germany (vine nursery Krapp). Cuttings of ‘Fleurtai’ (*Rpv12*) [61] were provided from Viva Cooperativi Rauscedo (Italy) obtained from their vineyard. The plant material of this study has been identified and certified by Mr. Neser (Agricultural chamber of Palatinate, Neustadt, Germany) and is deposited in the herbarium of the Julius Kühn-Institut (Bundesforschungsinstitut für Kulturpflanzen, Geilweilerhof, Siebeldingen, Germany). Potted grapevines were grown under greenhouse conditions (22 °C/day, 18 °C/night; 50% humidity). The *Plasmopara viticola* isolates that are virulent on *Rpv3–1* genotypes or avirulent on resistant genotypes were collected as described previously [32]. The *P. viticola* VB-isolate, which overcomes *Rpv12-* and *Rpv3-*mediated resistance was isolated in Soyhières (Switzerland) in an isolated vineyard of Valentin Blattner (VB), in which an *Rpv3*-cultivar was cultivated over several years, followed by a *Rpv12*-cultivar. Inadequate plant protection treatments were performed in this vineyard. These isolates were designated *avrRpv*^*+*^ (avirulent on all resistant cultivars), *avrRpv3ˉ* (virulent on *Rpv3–1-*genotypes) and *avrRvp12*^*−*^*/3*^*−*^ (virulent on *Rpv12*- and/ or *Rpv3–1-*genotypes), followed the classification used by Casagrande et al. [13] and Eisenmann et al. [32]. Propagation of isolates was performed as described by Malacarne et al. [45].

### Leaf disc infection

Leaf disc infection assay, with infection suspension containing 50,000 sporangia ml^− 1^ or dH_2_O as control, was performed as described in Eisenmann et al. [32]. For sporulation experiments four 10 μl droplets, for histochemical studies one 20 μl and for HPLC experiments one 40 μl droplet of zoospore suspension were placed on the abaxial leaf surface. Leaves were sampled from potted greenhouse grapes, which were grown from end of April until the beginning of June. The fourth and fifth leaf from the apex were used for sampling leaf discs. Experiments were performed in June to have optimum infection conditions.

### Phenotypic assessment of plant resistance to *Plasmopara viticola* isolates

For the evaluation of each *P. viticola* isolate, a total of 21 leaf discs were cut from leaves sampled from three individual plant replicates (´Müller-Thurgau´, ´Riesling´, ´Regent´, ´Cabernet Blanc´, ´Merlot Khorus´, ´Fleurtai´, ´Sauvignac´, ´Soreli´ and ´Muscaris´). Leaf discs were infected and incubated as described in Eisenmann et al. [32]. The pathogen development was macroscopically scored at 6 days post inoculation (dpi) (Canon EOS800D with a Sigma 105 mm F2.8 EX DG MACRO OS objective). The number of sporangia produced per leaf disc at six dpi was quantified by counting and used to describe the degree of *P. viticola* infection [76]. Presented are the mean values of three independent experiments. Averages were used for Kruskal-Wallis and Steel-Dwass-Critchlow-Fligner test to analyse the data. The software used for statistical analysis was XLSTAT 2019.3.2, Add-On for Microsoft Excel®.

### Histochemical studies

For all histochemical studies, a total of three leaf discs from three individual plants in three independent experiments (*n* = 9) were inoculated, incubated as previously described [32]. To observe the development of *P. viticola* within leaves aniline blue staining was performed according to Hood and Shew [77]. Samples were collected at 24, 48, 72 and 96 hpi and documented with an epifluorescence microscope (ZEISS Axio Scope.A1; Kübler HXP-120C lighting device; camera: Axiocam MRc; filter set: Zeiss 05). Cell death was detected by trypan blue staining at 6, 8, 12, 24, 28 and 32 hpi as described by Koch and Slusarenko [78]. Stained leaf discs were analyzed with a ZEISS Axio Scope.A1 microscope with a Zeiss Axiocam MRc camera. Area of cell death was measured with the software Fiji [79]. The cell death areas were measured using the ROI (region of interest) manager of the Fiji software. Five images of different leaf discs were taken at 20x magnification. Each cell death area was outlined by hand and each ROI was calculated by the program. The mean value in mm^2^ is displayed. Error bars show the standard deviation. Diaminobenzidine staining was used to detect hydrogen peroxide formation in *P. viticola* infected leaf discs. The staining method was adapted from Daudi and O’Brien [80]. Infected leaf discs were sampled at 0, 2, 4, 6, 8, 10, 12, 24, 28 and 32 hpi. Therefore, they were placed 1 h before sampling time point at a distance of 6.5 cm under a LED-lamp (PHILIPS GreenPower LED production module deep red/white 150 50–60 Hz and 35 W) with ~ 672.5 μmol s^− 1^ m^− 2^ (measured with LI-250A light meter; sensor: LI-core Qantum) to open the stomata. The infection droplet was removed before staining them with diaminobenzidine staining solution (3, 3′-diaminobenzidin-tetrahydrochlorid 1 mg ml^− 1^ in water, TWEEN 20 (0.05% (v/v)), 200 mM Na_2_HPO_4_, Break-Thru (0.1% (v/v)). Each leaf disc was vacuum infiltrated twice 30–60 s followed by incubation for 30 min in the dark. Destaining (ethanol: acidic acid: glycerine (3:1:1)) was performed for 15–30 min in a boiling water bath. Microscopic analysis was performed with ZEISS Axio Scope.A1 microscope with a Zeiss Axiocam MRc camera. For all histochemical studies Zen blue software was used.

### Measuring of *trans-*resveratrol

One experiment consists of 10 individual leaf discs from 5 individual plants (´Müller-Thurgau´, ´Regent´, ´Fleurtai´ and ´Merlot Khorus´). Samples per treatment (infected/mock) were pooled together at 0, 6, 8, 12, 24, 48 hpi (*n* = 10) and repeated three times (*n* = 3), except of ´Fleurtai´ (*n* = 2). Prior to freezing in liquid nitrogen the outer 2 mm wounded edge of each leaf disc was removed to reduce potential effects of the wounding caused by cutting of the discs in stilbene production. The leaf discs were spread on a petri dish, infected and stored as described above. Stilbenes were extracted as described by Höll et al. [81]. The extracts were analyzed with HPLC (Jasco 4000er, Jasco AS-4150 autosampler, Jasco PU-4180 pump). Extracts were separated with a reversed phase column (Gemini-NX 3 μm, C18 110 Å, LC column 150 × 4.6 (Phenomenex® LTD, Aschaffenburg, Germany) protected by a pre-column. *Trans*-resveratrol levels were measured with a photo diode array detector (Jasco MD-4010) at 320 nm excitation wavelength. Separation was performed with a gradient of buffer A (10 mmol KH_2_PO_4_; 5% (v/v) acetonitrile (HPLC grade); 95% (v/v) water (HPLC grade); pH adjusted with 85% H_3_PO_4_ to 1.5) to buffer B (10 mmol KH_2_PO_4_; 50% (v/v) acetonitrile (HPLC grade); 50% (v/v) water (HPLC grade); pH adjusted with 85% H_3_PO_4_ to 1.5). The gradient conditions were 0 min, 90% A; 7 min, 66% A; 12 min, 49% A; 17 min, 68% B; 22 min, 100% B; 28 min, adjustment to initial conditions. The column was maintained at 25 °C and the flow rate was 1 ml min^− 1^. The emission detector was set to a wavelength range of 200–650 nm. Data acquisition and processing was performed with ChromNav Software (Jasco). For calculation of *trans*-resveratrol concentrations a calibration curve prepared from commercially available *trans*-resveratrol standard (Phyto-Lab) was used. The level of *trans*-resveratrol (ng g^− 1^ fresh weight (FW)) was normalized against untreated control. Results of three independent experiments are shown**.**

### Field trials

Disease evaluation in the field was performed in a randomised block design of 25 grapevines per replicate with four replicates per treatment. For each rating block, 25 leaves and 25 grapes were evaluated randomly from both sides of the row. The location of rating blocks differed between all experimental years and was repositioned annually. Fungicide application trials using `Sauvignac` and `Cabernet Blanc` were undertaken at three commercial vineyards located in Rhineland-Palatinate, Germany. The fungicide treatment plan, as follows, was performed by the vineyard owners (Additional file 3). The time point of treatment was predefined to the grapevine development (BBCH-scale) according to Lorenz et al. [82], but the use of a specific fungicide was not requested. Identical treatment variants were chosen for both grape cultivars. In treatment variant four the first treatment took place during early leaf development (BBCH 15), followed by two treatments during flowering, (BBCH 57–60 & BBCH 69) and a final treatment when berries reach pea-size (BBCH 75–77) (Additional file 3). The second variant was treated during flowering (BBCH 57–59 & BBCH 69) and an untreated variant was monitored as a control to classify and compare annual natural infection pressure (Additional file 3). The susceptible cultivar `Kerner` was treated conventionally, independent from the treatment plan, by the vineyard owner. In 2016 *P. viticola* infection pressure was extremely high, therefore the study was stopped by additional applications of a fungicide at BBCH 71 to ensure plant health. The disease severity scheme of the European Plant Protection Organization (EPPO) was modified and used to assess disease severity [83]. Only seven symptom grades were used: 0%, (no symptoms); < 5%, < 10%, < 25%, < 50%, < 75% and ≤ 100% infection. For the evaluation of disease incidence the same percentage scale was used. Average disease incidence and severity from different locations were used for statistical analysis. Due to extreme conditions or deficiencies in single locations, not all averages for all three locations during the various years of trials were compiled (2016: (n (grapes/application number) = 200; 2017 and 2018: (n (grapes/application number) = 300) and ´Kerner´ 2016–2018: *n* = 100. Kruskal-Wallis and Conover-Iman test were used to analyse the data. The software used for statistical analysis was XLSTAT 2019.3.2, Add-On for Microsoft Excel®.

## Supplementary Information

**Additional file 1. **Trypan blue staining of susceptible cultivar upon *P. viticola* (*avrRpv*^*+*^) infection and mock inoculation (water). (A) Trypan blue staining of susceptible genotype (´Müller-Thurgau´) upon infection with *avrRpv*^*+*^ after 8, 12 and 32 hpi, st, stomata; z, encysted zoospore (scale bar correspond to 20 μm). (B) Trypan blue staining of susceptible genotype (´Müller-Thurgau´) after mock inoculation and upon infection with *avrRpv*^*+*^ after 6 dpi (scale bar correspond to 100 μm).

**Additional file 2. **Trypan blue and diaminobenzidine staining analysis of all genotypes upon mock inoculation (water). (A) Trypan blue staining of susceptible genotype (´Müller-Thurgau´) and resistant *Rpv3-* (´Regent´), *Rpv12-* (´Fleurtai´), *Rpv12/Rpv3-* (´Sauvignac´) and *Rpv10-*genotype (´Muscaris´) after mock inoculation at 32 hpi (scale bar correspond to 20 μm), st, stomata. (B) Diaminobenzidine staining of susceptible genotype (´Müller-Thurgau´) and resistant *Rpv3-* (´Regent´), *Rpv12-* (´Fleurtai´) *Rpv12/Rpv3-*genotype (´Sauvignac´) after mock inoculation at 24 hpi (scale bar correspond to 50 μm).

**Additional file 3.** Plant protection treatment plan. Fungicide treatments were implemented according to the grapevine developmental stage (BBCH). The different variants with four, two or zero treatments were evaluated during growing seasons. (+) Fungicide treatment at the appropriate BBCH stage was applied. (−) Absence of fungicide application.

**Additional file 4. ***Plasmopara viticola* disease incidence and severity in leaves (BBCH 71 – 2016; BBCH 75 – 2017, 2018). Disease incidence (black bars) and disease severity (grey bars) were quantified over three seasons in the grapevine cultivars ´Sauvignac´ (*Rpv12/Rpv3*) and ´Cabernet Blanc´ (*Rpv3*), differing in the number of fungicide applications during the season. The bars show mean values for two different locations in 2016 (n (grapes/application number) = 200) and from three different locations in 2017 and 2018 (n (grapes/application number) = 300). Error bars show standard error (SEM); no infections detected (n.d.). Kruskal-Wallis and Conover-Iman test was used to compare the disease incidence (A, B, C) and severity (a, b, c) for each year, *p* < 0.05.

**Additional file 5. **Comparison of *Plasmopara viticola* development in leaves of susceptible and resistant cultivars. Development of (A) *avrRpv12*^*−*^*/3*^*−*^ and (B) *avrRpv3*^*− *^*P. viticola* isolates on leaf discs of the susceptible genotype (´Müller-Thurgau´) and resistant *Rpv3-* (´Regent´), *Rpv12-* (´Fleurtai´), *Rpv12/Rpv3-* (´Sauvignac´) and *Rpv10-*genotype (´Muscaris´), were evaluated using UV epifluorescence after aniline blue staining at 96 hpi. Arrows indicate infection structures of *P. viticola.* Images are representative of three biological replicates in three independent experiments. Scale bars correspond to 100 μm.

**Additional file 6. **Induction of cell death at *Plasmopara viticola* infection sites. (A) *avrRpv12*^*−*^*/3*^*−*^ and (B) *avrRpv3*^*−*^*P. viticola* were used to inoculate leaf discs of susceptible genotype (´Müller-Thurgau´) and resistant *Rpv3-* (´Regent´), *Rpv12-* (´Fleurtai´), *Rpv12/Rpv3-* (´Sauvignac´) and *Rpv10-*genotype (´Muscaris´), samples were taken at 8 hpi and 12 hpi. Yellow asterisks indicate trypan blue stained cells showing cell death, st, stomata; z, encysted zoospore. Images are representative of three biological replicates in three independent experiments. Scale bars correspond to 20 μm.

## Data Availability

The datasets supporting the conclusions of this article are included within the article and its additional files.

## Abbreviations

*avrRpv*^+^: Avirulent on all resistant cultivars
*avrRpv3ˉ*: Virulent on *Rpv3-*genotypes
*avrRvp12*^−^*/3*^−^: Virulent on *Rpv12-* and/ or *Rpv3-*genotypes
DAB: Diaminobenzidine
DAMPs: Danger-associated molecular patterns
dpi: Days post inoculation
ETI: Effector-triggered immunity
FRC: Fungus-resistant grapevine cultivars
H_2_O_2_: Hydrogen peroxide
HR: Hypersensitive response
hpi: Hours post inoculation
NB-LRR: Nucleotide-binding domains and leucine-rich-repeats
PAMPs: Pathogen associated molecular pattern
PRRs: Pattern recognition receptors
PTI: PAMP-triggered immunity
QTL: Quantitative trait loci
ROS: Reactive oxygen species
*Rpv*: Resistance to *Plasmopara viticola*
