# Supplementary figures and images for: Grapevine Rpv3-, Rpv10- and Rpv12-mediated defense responses against Plasmopara viticola and the impact of their deployment on fungicide use in viticulture
Source: BMC Plant Biol. 2021 Oct 14;21:470. doi: 10.1186/s12870-021-03228-7 (PMC8515710; doi:10.1186/s12870-021-03228-7)

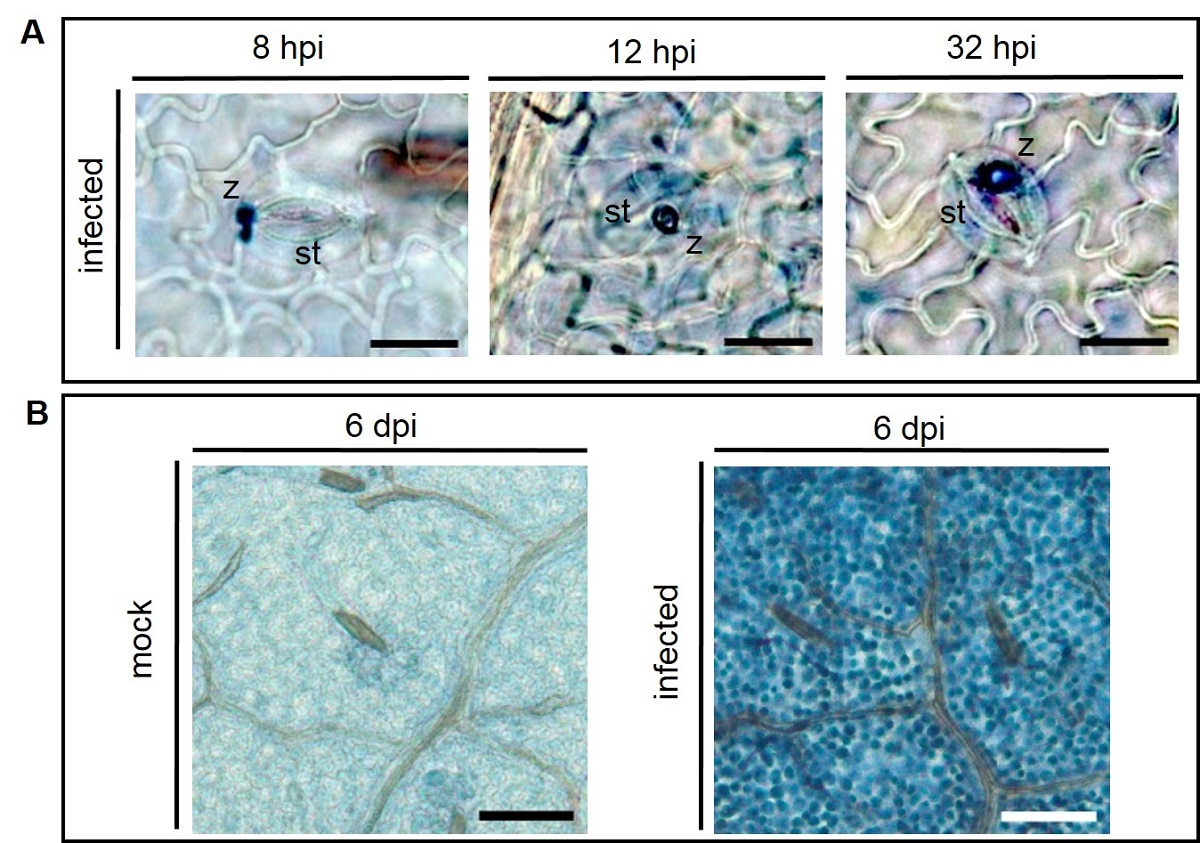

Supplement: Supplementary file 1 — Additional file 1. Trypan blue staining of susceptible cultivar upon P. viticola (avrRpv+) infection and mock inoculation (water). (A) Trypan blue staining of susceptible genotype (´Müller-Thurgau´) upon infection with avrRpv+ after 8, 12 and 32 hpi, st, stomata; z, encysted zoospore (scale bar correspond to 20 μm). (B) Trypan blue staining of susceptible genotype (´Müller-Thurgau´) after mock inoculation and upon infection with avrRpv+ after 6 dpi (scale bar correspond to 100 μm). [file 12870_2021_3228_MOESM1_ESM.jpg]

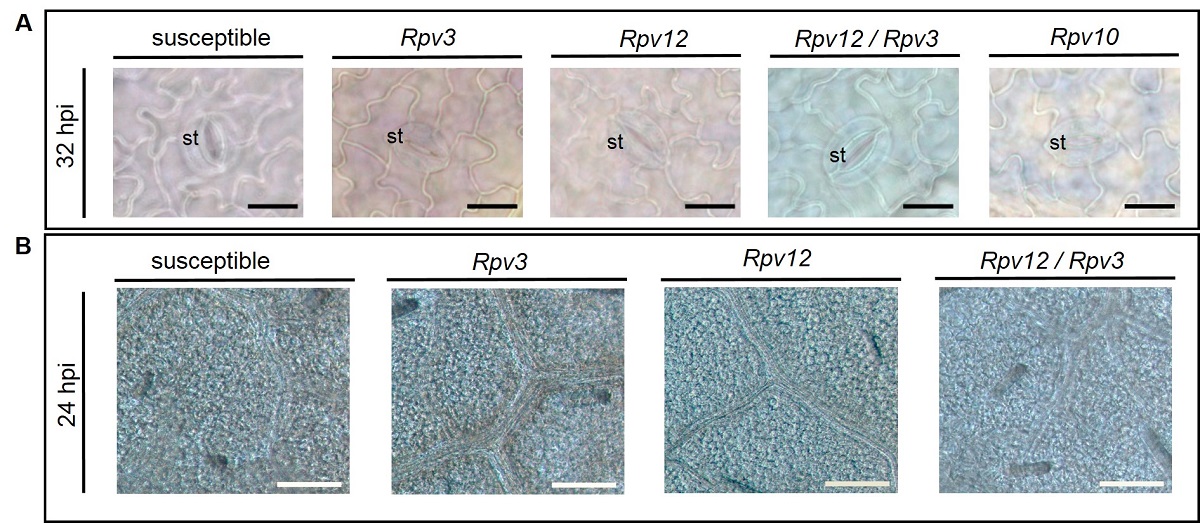

Supplement: Supplementary file 2 — Additional file 2. Trypan blue and diaminobenzidine staining analysis of all genotypes upon mock inoculation (water). (A) Trypan blue staining of susceptible genotype (´Müller-Thurgau´) and resistant Rpv3- (´Regent´), Rpv12- (´Fleurtai´), Rpv12/Rpv3- (´Sauvignac´) and Rpv10-genotype (´Muscaris´) after mock inoculation at 32 hpi (scale bar correspond to 20 μm), st, stomata. (B) Diaminobenzidine staining of susceptible genotype (´Müller-Thurgau´) and resistant Rpv3- (´Regent´), Rpv12- (´Fleurtai´) Rpv12/Rpv3-genotype (´Sauvignac´) after mock inoculation at 24 hpi (scale bar correspond to 50 μm). [file 12870_2021_3228_MOESM2_ESM.jpg]

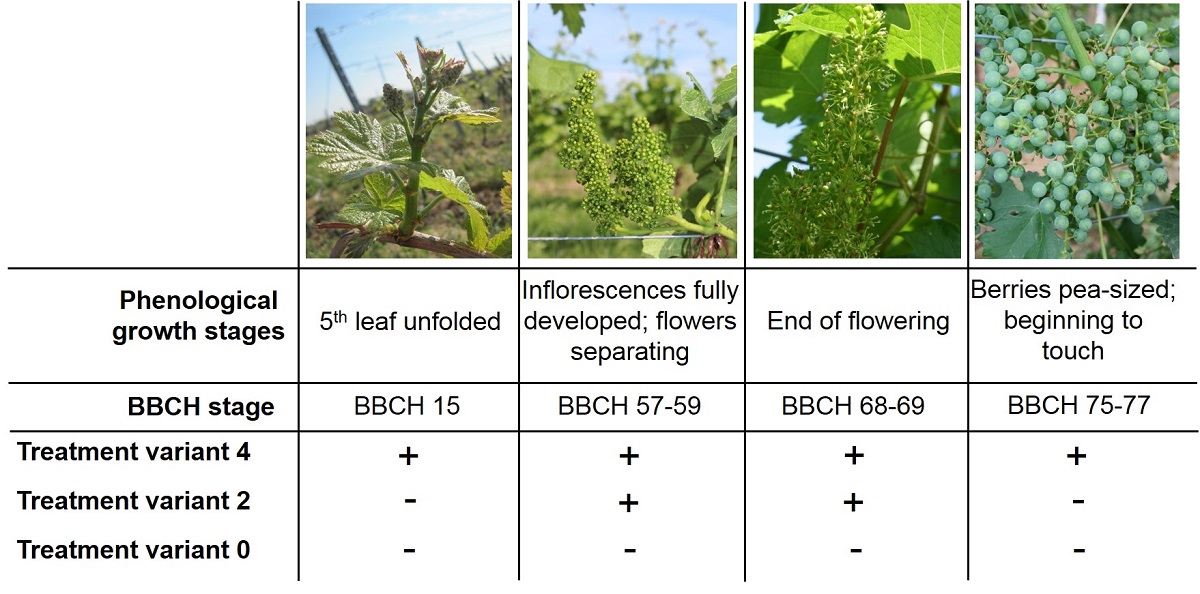

Supplement: Supplementary file 3 — Additional file 3. Plant protection treatment plan. Fungicide treatments were implemented according to the grapevine developmental stage (BBCH). The different variants with four, two or zero treatments were evaluated during growing seasons. (+) Fungicide treatment at the appropriate BBCH stage was applied. (−) Absence of fungicide application. [file 12870_2021_3228_MOESM3_ESM.jpg]

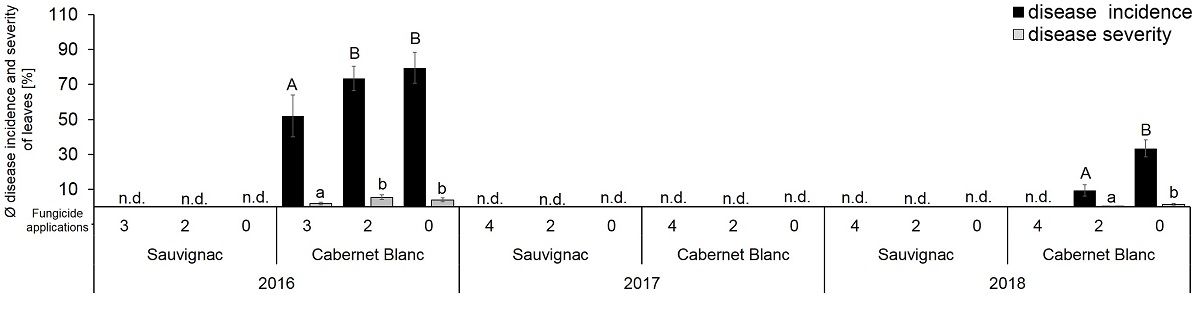

Supplement: Supplementary file 4 — Additional file 4. Plasmopara viticola disease incidence and severity in leaves (BBCH 71 – 2016; BBCH 75 – 2017, 2018). Disease incidence (black bars) and disease severity (grey bars) were quantified over three seasons in the grapevine cultivars ´Sauvignac´ (Rpv12/Rpv3) and ´Cabernet Blanc´ (Rpv3), differing in the number of fungicide applications during the season. The bars show mean values for two different locations in 2016 (n (grapes/application number) = 200) and from three different locations in 2017 and 2018 (n (grapes/application number) = 300). Error bars show standard error (SEM); no infections detected (n.d.). Kruskal-Wallis and Conover-Iman test was used to compare the disease incidence (A, B, C) and severity (a, b, c) for each year, p < 0.05. [file 12870_2021_3228_MOESM4_ESM.jpg]

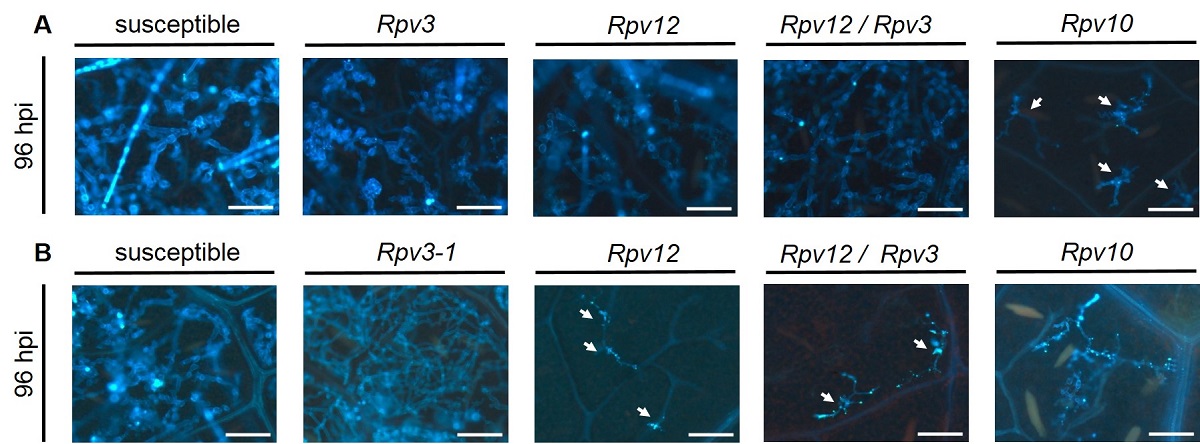

Supplement: Supplementary file 5 — Additional file 5. Comparison of Plasmopara viticola development in leaves of susceptible and resistant cultivars. Development of (A) avrRpv12−/3− and (B) avrRpv3− P. viticola isolates on leaf discs of the susceptible genotype (´Müller-Thurgau´) and resistant Rpv3- (´Regent´), Rpv12- (´Fleurtai´), Rpv12/Rpv3- (´Sauvignac´) and Rpv10-genotype (´Muscaris´), were evaluated using UV epifluorescence after aniline blue staining at 96 hpi. Arrows indicate infection structures of P. viticola. Images are representative of three biological replicates in three independent experiments. Scale bars correspond to 100 μm. [file 12870_2021_3228_MOESM5_ESM.jpg]

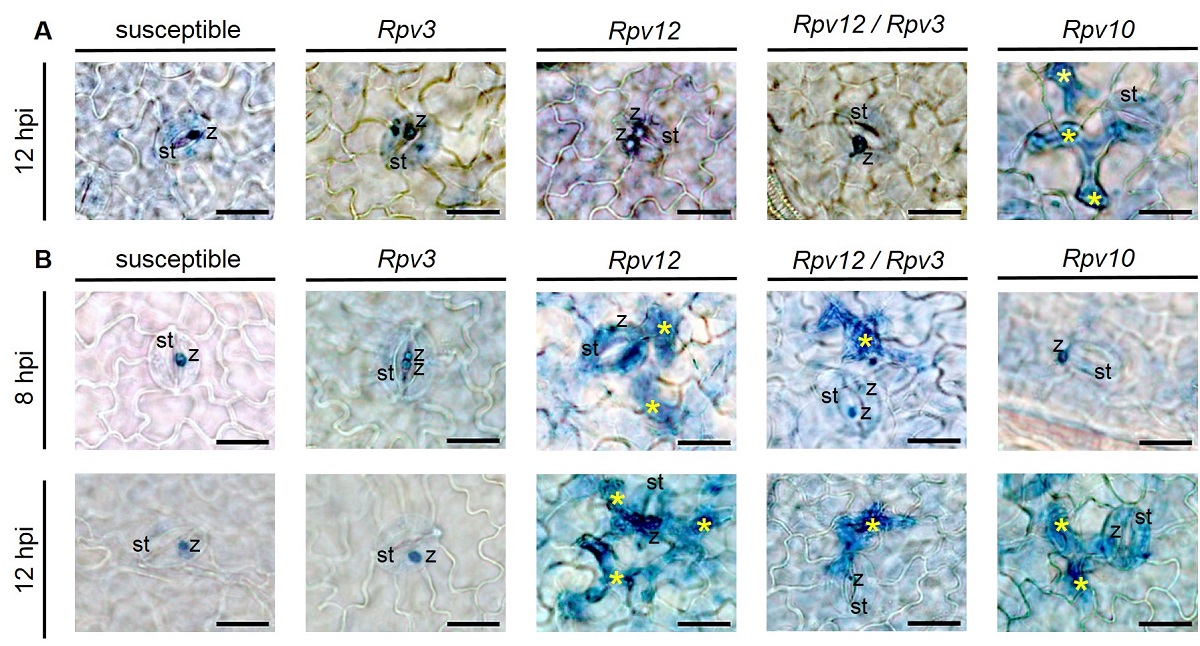

Supplement: Supplementary file 6 — Additional file 6. Induction of cell death at Plasmopara viticola infection sites. (A) avrRpv12−/3− and (B) avrRpv3−P. viticola were used to inoculate leaf discs of susceptible genotype (´Müller-Thurgau´) and resistant Rpv3- (´Regent´), Rpv12- (´Fleurtai´), Rpv12/Rpv3- (´Sauvignac´) and Rpv10-genotype (´Muscaris´), samples were taken at 8 hpi and 12 hpi. Yellow asterisks indicate trypan blue stained cells showing cell death, st, stomata; z, encysted zoospore. Images are representative of three biological replicates in three independent experiments. Scale bars correspond to 20 μm. [file 12870_2021_3228_MOESM6_ESM.jpg]
